# Immunogenetics in hematopathology and hematology: why a common language is important

**DOI:** 10.1038/s41375-024-02260-4

**Published:** 2024-04-25

**Authors:** Kostas Stamatopoulos, Elspeth Bruford, Elias Campo, Marie-Paule Lefranc

**Affiliations:** 1 Institute of Applied Biosciences, Centre for Research and Technology Hellas, Thessaloniki, Greece; 2 Department of Molecular Medicine and Surgery, Karolinska Institute, Stockholm, Sweden; 3 Department of Haematology, University of Cambridge, Cambridge Biomedical Campus, Cambridge, UK; 4 European Molecular Biology Laboratory, European Bioinformatics Institute, Wellcome Genome Campus, Cambridge, UK; 5 Hematopathology Section, Pathology Department, Hospital Clínic, University of Barcelona, Barcelona, Spain; 6 Molecular Pathology of Lymphoid Neoplasms Group, Institut d’Investigacions Biomèdiques August Pi i Sunyer (IDIBAPS), Barcelona, Spain; 7 IMGT®, the international ImMunoGeneTics information system®, Laboratoire d’ImmunoGénétique Moléculaire (LIGM), Institut de Génétique Humaine (IGH), Centre National de la Recherche Scientifique (CNRS), Université de Montpellier, Montpellier, France

**Keywords:** Haematological cancer, Adaptive immunity

## Immunogenetics—key to understanding and managing lymphoid malignancies

A defining feature of the immune system concerns the generation of a remarkably diverse repertoire of antigen-specific lymphocytes, each carrying a unique antigen receptor: the immunoglobulin (IG) or antibody and the T cell receptor (TR) on the surface of any mature B or T cell, respectively [1, 2]. The IG are composed of four polypeptide chains, two identical heavy (IGH) chains and two identical light chains, kappa (IGK) or lambda (IGL) [1]. The TR are composed of two chains, alpha (TRA) and beta (TRB), or gamma (TRG) and delta (TRD), defining two types of T cells [2].

IG and TR genes are assembled in the early stages of B and T cell development, respectively, with V-(D)-J or V-J rearrangements of distinct variable (V), diversity (D) [only in the IG heavy (IGH) locus and the TR beta (TRB) and delta (TRD) loci, respectively] and joining (J) genes [3], generating combinatorial diversity. During V-(D)-J rearrangement, diversity is further increased by the N-diversity which results from the exonuclease trimming at the 3’ end of the V, both ends of the D, and 5’ end of the J coding regions, followed by the random addition of non-templated nucleotides (N) by the DNA nucleotidylexotransferase (DNTT, TdT, terminal deoxynucleotidyl transferase) before gene ligation at the V-(D)-J junctions. A third source of diversity for the IG concerns the somatic hypermutations (SHM) which occur later during B cell differentiation in the rearranged V-(D)-J genes [4]. Further, during the transcription in B or T cells, the rearranged V-(D)-J gene which encodes the V domain is spliced to a C gene that encodes the C region. Finally, the random pairing of heavy/light and gamma/delta or alpha/beta chains in the IG or TR, respectively, represents an additional source of diversity for the antigen receptors.

Human naïve T cells have been estimated to express 10 × 10^6^ distinct TR beta (TRB) chains, each of them randomly paired with one of 100 distinct TRA chains, giving rise to a theoretical 10^9^ different, unique TR. Hence, naïve T cells have the potential to respond to a very wide range of antigens whether these derive from pathogens (exogenous antigens) or the host (autoantigens). Unsurprisingly, the diversity of antigen-experienced (particularly, memory) T cells is more restricted; however it allows rapid responses to a future encounter with the selecting antigens. In B cells, thanks to the combined effects of combinatorial diversity, modifications at the junctions of the recombining genes and SHM, the BcR IG gene repertoire is immense. In fact, according to recent estimates, the BcR IG repertoire could be in the region of 10^16^–10^18^ unique possibilities [5–7].

Lymphoid malignancies comprise tumors of vastly different cells of origin, biological background and clinical presentation and course [8]. That said, a unifying theme for all lymphoid malignancies is their antigen receptor gene rearrangement, which constitutes a unique molecular identity of the respective expanded lymphoid clone [9]. More particularly, since IG and TR gene rearrangements begin at the very early stages of lymphoid differentiation, they are already present when a lymphoid cell transforms, and hence can be detected in almost all malignancies of immature and mature lymphoid cells. These rearrangements are easily identifiable at diagnosis and independent of clinical stage or other biomarkers. Moreover, with certain exceptions (most notably in precursor B cell acute lymphoblastic leukemia) [10], they tend to remain stable over time and are not influenced by, and do not reflect, disease evolution, excepting sequence variation that may be introduced within the IG genes by ongoing SHM due to continuous interactions with antigen(s) [9, 11].

Ample evidence supports that the study of antigen receptor gene rearrangements in lymphoid malignancies provides a key to understanding their ontogeny; assists in establishing the correct diagnosis by documenting clonality; offers critical prognostic and predictive information (e.g. the determination of the SHM status of the clonotypic rearranged IG heavy variable (IGHV) genes in chronic lymphocytic leukemia); and aids in monitoring the response to treatment through the assessment of measurable residual disease in several lymphoid neoplasms [9].

## The language of immunogenetics: the need of consistency

### IMGT-ONTOLOGY: standardization of immunogenetic nomenclature

One of the most important aspects in the implementation of immunogenetics in both research and routine diagnostics concerns standardization of terminology. IMGT®, the international ImMunoGeneTics information system® (https://www.imgt.org), was at the origin of immunoinformatics, a new science at the interface of immunogenetics and bioinformatic^s^ [12].

IMGT-ONTOLOGY includes a controlled vocabulary and annotation rules which are indispensable to ensure accuracy, consistency and coherence of immunogenetics data with scientific knowledge and, most particularly, in clinical applications [13]. The IMGT concepts and standards are needed more than ever in the era of immunogenetics using next generation sequencing (NGS), which has raised the major challenge of analyzing millions of rearranged IG and TR gene sequences with inherent complexity, variability and mutational capacity [14]. This challenge extends from raw data processing and V-(D)-J gene assignment and clonotype identification to meta-analysis and clonotype comparisons between different lymphoid individuals/diseases.

### IMGT-ONTOLOGY classification concepts: locus and gene names and symbols

**Fig. 1:**
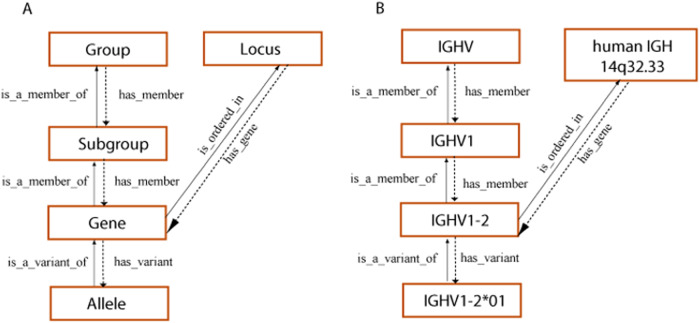
Concepts of classification for IMGT gene and allele nomenclature (CLASSIFICATION axiom) [16, 26]. **A** Hierarchy of the concepts of classification and their relationships. The definition of the reciprocal relations between concepts can be read, from one concept to the other, either ascending the hierarchy (solid arrows) or descending the hierarchy (dotted arrows). **B** Examples of concept instances for each concept of classification. The concept instances are associated with an instance of the “Taxon” concept, and more precisely for the “Gene” and “Allele” concepts, to an instance of the “Species” concept (here, *Homo sapiens*). The “Locus” concept is a concept of localization (LOCALIZATION axiom). It is shown with a reciprocal relationship to the “Gene” concept. (With permission from M-P. Lefranc and G. Lefranc, LIGM, Founders and Authors of IMGT^®^, the international ImMunoGeneTics information system^®^, https://www.imgt.org).

A major additional challenge concerns the “vocabulary” and “language” of immunogenetics in the specific field of antigen receptors. IMGT has systematically worked on this [15–18] by providing a standardized nomenclature for immunoglobulin (IG) and T cell receptor (TR) genes in human and other vertebrates that is based on the concepts of classification (locus, group, subgroup, gene, allele) of the IMGT-ONTOLOGY (‘CLASSIFICATION’ axiom) (Fig. 1), and follows as closely as possible the HUGO Gene Nomenclature Committee (HGNC) guidelines [19]. This was applied in 1988 for the human IGL and IGH loci, and in 1989 for the variable (V), joining (J) and constant (C) genes of the newly identified human TRG locus [20, 21]: gene names/symbols in capital letters, no Greek letters, no commas, no periods/full stops (hyphens are accepted, as they are for the HLA genes). IG and TR genes and alleles are not italicized in publications.

The three first letters indicate the locus: IGH, IGK, IGL, TRA, TRB, TRG, TRD [20–23]. The fourth letter is V (for a V-GENE), D (for a D-GENE), J (for a J-GENE) or C (for a C-GENE) (except for IGH, see below). The subsequent number(s) and/or letter(s) allow, if necessary, unambiguous identification of the gene, a single number or letter being used whenever possible: IGKC, TRGV9, TRGVA, TRAJ4 etc. For the IGH locus, the constant genes are designated by the letter (and number suffix) corresponding to the encoded isotype (IGHM, IGHD, IGHG3,…), instead of using the letter C. For the more complex situation where genes have not yet been localized and have to be named according to their subgroup (for V) or cluster (for D and J), a temporary designation is used in which the Arabic number (for the subgroup or cluster) is followed by the letter S (for subgroup or sequential, respectively), followed by the number of the gene in the subgroup or cluster. Orphon genes (OR) located outside the main chromosomal locus are designated by the IMGT four letter gene designation, followed by a number for the subgroup (if known) or between parentheses a Roman numeral for the clan, a slash, “OR”, the chromosome number (if known), a hyphen and a specific gene number and/or letter. Examples: IGKV2/OR2-1, IGHD1/OR15-1a, IGKV1/OR-1, IGHV(III)/OR16-25.

IMGT gene names (or symbols) and IMGT gene definitions (or full names) for all the human IG and TR genes were approved by the HGNC in 1999 and listed originally in the Genome DataBase (GDB), and at the NCBI’s LocusLink, then Entrez Gene, and now Gene (at www.ncbi.nlm.nih.gov/gene), as well as in the HGNC’s database at www.genenames.org. Note that in the HGNC symbols for IG and TR genes the slashes and parentheses are omitted. Otherwise, the IG and TR gene names/symbols are identical between IMGT and HGNC. All the IMGT gene definitions (including those with slashes and parentheses) have been endorsed as official gene names by HGNC (“approved name”) and NCBI Gene (“official full name”).

### How to report chromosomal translocations involving IG and TR loci

Under normal circumstances IG and TR names/symbols are not italicized. The single tolerated exception to these consensus rules concerns the use of italics for the names/symbols of IG and TR loci participating in chromosomal rearrangements, described in specialist databases and/or journals following the IMGT and HGNC recommendations. The use of italics indicates that an IG or TR locus is ‘**translocated**’, i.e affected by an internal or external (beyond the locus 5’ or 3’ border) breakpoint, and therefore does not represent the normal IG or TR locus [1, 2]. This unique exception for IG and TR loci was agreed between IMGT and HGNC following the publication of the HGNC’s recommendation to use a double colon (::) as the separator between gene symbols in translocations generating fusion genes [24]. Subsequently, the 5^th^ edition of the WHO classification of hematological neoplasms (WHO-HAEM5) has adopted this separator for translocations which involve conventional genes and IG and TR loci in hematological neoplasms, also italicizing these genes and IG and TR loci (e.g *IGH*::*MYC* and *IGH*::*CCND1*) [25]. While the WHO-HAEM5 “follows the HGNC guidelines regarding italicizing immunoglobulin (*IGH, IGK, IGL*) and T cell receptor (*TRA, TRB, TRD, TRG*) loci”, note that this italicization is an exception limited only to ‘translocated’ IG or TR loci and only in this context of IMGT and HGNC recommendations.

## Concluding remarks

Immunogenetics has an undisputed role in both the research and the management of lymphoid malignancies. Hence, the major challenge is how to meet the highest standards and ensure consistency that translates into obvious benefits for both advancing research and providing accurate diagnostic information for managing patients. In this context, speaking a common language ensures that we understand each other and communicate scientific findings effectively while also minimizing misunderstandings and confusion.
